# A Correlation Study of DHA Intake Estimated by a FFQ and Concentrations in Plasma and Erythrocytes in Mid- and Late Pregnancy

**DOI:** 10.3390/nu9111256

**Published:** 2017-11-16

**Authors:** Yu-Bo Zhou, Hong-Tian Li, Leonardo Trasande, Lin-Lin Wang, Ya-Li Zhang, Ke-Yi Si, Man-Xi Bai, Jian-Meng Liu

**Affiliations:** 1 Institute of Reproductive and Child Health/Ministry of Health Key Laboratory of Reproductive Health, Peking University Health Science Center, 38 Xueyuan Rd., Beijing 100191, China; zhouyubo@bjmu.edu.cn (Y.-B.Z.); liht@bjmu.edu.cn (H.-T.L.); linlinwang@bjmu.edu.cn (L.-L.W.); zhangyl@bjmu.edu.cn (Y.-L.Z.); sikeyi0219@163.com (K.-Y.S.); 2 Department of Epidemiology and Biostatistics, School of Public Health, Peking University Health Science Center, 38 Xueyuan Rd., Beijing 100191, China; 3 Department of Pediatrics, School of Medicine, New York University, 227 East 30th Street, Room 735, New York, NY 10016, USA; Leonardo.Trasande@nyumc.org; 4 Wyeth Nutrition Science Center, 582 Wuzhong Rd., Shanghai 201103, China; manxi.bai@wyethnutrition.com

**Keywords:** docosahexaenoic acid, pregnant women, food frequency questionnaire, plasma, erythrocytes

## Abstract

Adequate docosahexaenoic acid (DHA) is essential for the optimal growth and development of the fetus. Maternal DHA content fluctuates during pregnancy. The correlation of DHA content with dietary intake might be varied over the course of pregnancy. We aimed to compare the dietary DHA intake, estimated by a DHA-specific semiquantitative food frequency questionnaire (FFQ) against its blood content, among mid- and late-term pregnant women. A total of 804 Chinese pregnant women completed the tailored FFQ and provided fasting venous blood samples. Dietary DHA intake (mg/day) in the previous month was calculated from the FFQ using Chinese Food Composition Table. DHA concentrations (weight percent of total fatty acids) in plasma and erythrocytes were measured by capillary gas chromatography. Spearman correlation coefficients (*r_s_*) between DHA intake and its relative concentrations were calculated. After adjustment for maternal age, pre-pregnancy body mass index, stage of pregnancy, parity, education level, ethnicity, and annual family income per capita, the correlation coefficients of DHA intake with its concentrations in plasma and erythrocytes were 0.35 and 0.33, respectively (*p* < 0.001). The correlations were relatively stronger among women in late pregnancy (*r_s_* = 0.44 in plasma and 0.39 in erythrocytes) than those in mid-pregnancy (*r*_s_ = 0.25 and 0.26). The significant correlations were consistently observed in subgroups stratified by regions, except for erythrocytes in women living in a coastland area. Multiple regression analyses also indicated significant positive linear correlations between DHA intake and its plasma or erythrocytes concentrations (*p* < 0.001). In conclusion, dietary DHA intake, estimated by the FFQ, was positively correlated with its concentrations in plasma and erythrocytes in Chinese pregnant women, especially for women in late pregnancy, with the exception of the erythrocytes of those living in a coastland area.

## 1. Introduction

Docosahexanoic acid (DHA, 22:6*n*-3), an essential *n*-3 long-chain polyunsaturated fatty acid, is an important structural component of brain grey matter and retinal phospholipids [1]. Sufficient DHA supply during pregnancy is critical for neural, visual and cognitive development of the growing fetus [2,3], and can significantly improve pregnancy outcomes, such as prolonging gestation duration by up to 2.9 days and increasing birth weight of the newborn by up to 172 g [4]. Dietary intake is the predominant source of DHA, due to the very limited conversion (<0.5%) from α-linolenic acid (18:3*n*-3) in vivo [5,6,7]. Therefore, the DHA status in biological specimens is sensitive to dietary intake. A precise assessment of DHA intake in pregnant women is becoming increasingly essential, given that the consensus suggests a dietary intake of at least 200 mg DHA per day for pregnant women [8,9,10].

Food frequency questionnaires (FFQ) are widely used to assess dietary DHA intake, especially in large-scale epidemiological studies [11]. Biomarkers in blood fractions (plasma may reflect intake over the past weeks and erythrocytes over the past months [12]) have been suggested as superior to FFQ, since they are objective, and their measurement errors are largely independent of those of the FFQ [13,14]. Studies have corroborated a positive correlation between the DHA content in the blood of pregnant women, with DHA intake measured by FFQs [15,16,17,18,19,20,21]. Yet, most of these studies were conducted in countries with higher rates of fish consumption [15,16,17,19], while similar studies in China are sparse [21]. Since the FFQ is very sensitive to cultural and dietary practices [22] and dietary patterns in China are changing over time [23], it is of great interest to develop a valid FFQ compatible with the diet of Chinese pregnant women and able to estimate their DHA intakes. Moreover, a wide range (0.21–0.63) of correlation identified from those studies was especially notable, with relatively a weaker correlation in mid-pregnancy (correlation coefficients were 0.21 for plasma and 0.25 for erythrocytes) [15,18] while stronger in late pregnancy (corresponding coefficients were 0.63 and 0.61) [16,19]. Because maternal DHA status changes over the course of pregnancy [24,25], the correlation between DHA concentration and dietary intake might be varied in different stages of pregnancy. However, to our knowledge, no study has been conducted to examine the degree to which correlations evolve during pregnancy using the same FFQ.

In the present study, we aimed to evaluate correlations between DHA intake, estimated by FFQ, with blood concentrations among Chinese pregnant women, and to determine whether the correlation held constant for mid- and late-term pregnant women.

## 2. Materials and Methods

### 2.1. Subjects

This was a cross-sectional study in women, at 15–19 (mid-pregnancy) or 37–41 gestational weeks (late pregnancy), identified from hospitals located in Weihai (coastland), Yueyang (lakeland) and Baotou (inland) city, between May and July of 2014. Eligible women were healthy, aged 18–35 years, local permanent residents, expected to deliver a singleton birth, and without severe heart, liver, kidney or lung diseases, or serious mental illnesses. Women were not included if they were allergic to fish, shrimp, shellfish or other DHA-rich foods, or they had participated in other research projects in the past 30 days, or they were judged to have severe vomiting by their obstetrician after 16 weeks of gestation. A total of 838 women were actually recruited, among whom, 17 women aged >35 years were excluded and 17 were further excluded since they were neither at 15–19, nor at 37–41 gestational weeks (Figure S1). The 804 available for the final analyses were all those who completed dietary assessments and provided a fasting blood sample. The study protocol was approved by the Institutional Review Board/Human Subjects Committee at Peking University Health Science Center (IRB00001052-14012; date of approval: 22 April 2014). The principles of the Declaration of Helsinki were applied, with all women being provided with informed consents and clearly informed of their right to withdraw from the study without giving any reason.

### 2.2. Dietary Assessment

Dietary information in the previous month was collected using a validated, electronic, tablet-based, DHA-specific, semiquantitative FFQ, named the DHA Intake Evaluation Tool [26]. Briefly, the FFQ included seafood (75 items, such as mackerel, lobster and crab), freshwater food (38 items, such as carp, shrimp and river crab) and mutton. DHA-containing supplements (DHA fortified milk powder, fish oil capsules, etc.) were also taken into account. For each item, a standard portion size was specified, and the women were asked the frequency, on average, of consumption of that specified amount during the previous month. The consumption frequency had 12 options, which varied from “once per month” to “three times per day”. The edible portion size had nine choices, ranging from 25 g to 250 g. The dietary DHA intake in the previous month was calculated by multiplying the frequency of each food consumed, by the average amount consumed per unit of time, and by the DHA content of the food. The DHA content in the food was primarily based on the China Food Composition Table (CFCT, Version 2009) [27]. A product ingredient list of DHA supplements was also used to supplement the CFCT data. The FFQ was self-administered by pregnant women, with visual aids of pictures for each item of food and intake reference scales, which took them approximately 10 min to complete. The FFQ was checked for completeness by a trained obstetrician before submission.

A structured questionnaire was also used to collect demographic information of pregnant women, including maternal age, gestational age, parity, ethnicity, education level, annual family income per capita, height and weight before pregnancy.

### 2.3. Blood Sample Collection and Analysis

Five mL of venous blood was collected from all the women, after overnight fasting, into EDTA-containing tubes. The detailed procedures for blood processing and analysis have been described previously [28]. Briefly, within 4 h of blood drawing, blood samples were centrifuged, to obtain aliquots of plasma and erythrocytes. The erythrocytes were washed out with 0.9% (*w*/*v*) NaCl solution. These aliquots were stored at −20 °C at the local hospitals, for about 10 days, before being sent back on dry ice to the central laboratory, and then stored in a −80 °C freezer and analyzed within two months. Fatty acid concentrations were determined by Agilent 6890N capillary gas chromatography (Agilent Technologies, Palo Alto, CA, USA). Individual fatty acids were identified by comparison with reference standards. The data were recorded using the Agilent Open LAB software (Agilent Technologies, Santa Clara, CA, USA). The concentrations of DHA were expressed as relative concentrations (weight percent of total fatty acids, wt. %).

### 2.4. Statistical Analysis

As the dietary intake (*D* = 0.260) and concentrations of DHA (*D* = 0.059 for plasma and 0.061 for erythrocytes) had non-normal distributions, when tested by the Kolmogorov–Smirnov D test (*p* values < 0.01), non-parametric statistical methods were used. The medians and interquartile ranges (IQR) were computed for dietary DHA intakes and DHA concentrations in plasma and erythrocytes. Adjusted medians and adjusted IQRs were estimated, by fitting a quantile regression model [29], containing terms for maternal age (≤25, >25 to 30, and >30 years), pre-pregnancy body mass index (BMI; <18.5, 18.5 to <25, and ≥25 kg/m^2^), stage of pregnancy (mid-pregnancy and late pregnancy), parity (nulliparous, and multiparous), education level (middle school or less, high school, and college or above), ethnicity (Han and others), and annual family income per capita (<30,000, 30,000 to <50,000, 50,000 to <100,000, ≥100,000 Yuan, and missing). Arithmetic means and standard deviations (SD) were also calculated, to facilitate comparisons with estimates reported in previous studies. The statistical differences of DHA intake and blood concentrations between mid-term pregnant women and late-term pregnant women were tested with Mann–Whitney tests, and the differences across regions were tested using Kruskal–Wallis tests, followed by Bonferroni corrected Mann–Whitney tests for multiple comparisons.

Crude and partial Spearman rank correlation coefficients (*r_s_*), individually (Table S1) and simultaneously adjusted for maternal age, pre-pregnancy BMI, stage of pregnancy, parity, education level, ethnicity, and annual family income per capita, were calculated, to determine correlations between DHA concentrations in plasma or erythrocytes and dietary intake. The differences in correlation coefficients between women in mid-pregnancy and late pregnancy were tested using *t* tests with Fisher r-to-z transformation. Multivariate linear regression analyses were also undertaken, to detect linear trends of DHA concentrations across deciles of DHA intake. DHA concentration was entered into the model as a dependent variable; deciles of DHA intake, maternal age, pre-pregnancy BMI, stage of pregnancy, education level, ethnicity and annual family income per capita were entered as independent variables. Least squares means of DHA concentrations were calculated for each decile of intake. We calculated robust estimators of variance for these means, to allow for the deviation from assumption of normal distribution of dependent variables [30]. *P*-values for linear trends were calculated by assigning the median value of each decile of intake as a continuous variable. Agreement between the FFQ and blood specimen estimates was also assessed, by classifying women into quartiles of the DHA intake and concentration.

All analyses were performed with SPSS for Windows (version 20.0; SPSS Inc., Chicago, IL, USA). Differences between groups and correlations were considered statistically significant at *p* value < 0.05 (two-sided).

## 3. Results

### 3.1. Maternal Characteristics

The average age of pregnant women was (27.7 ± 3.0) years. A total of 82.7% of women were nulliparous, 73.5% with a normal pre-pregnancy BMI (18.5 to <25 kg/m^2^), 65.1% with college or above education, and 27.5% had an annual family income above 50,000 Yuan per capita. The proportions of women residing in coastland, Lakeland and inland regions were 32.7%, 33.2% and 34.1%, respectively. Of the 804 pregnant women who completed the FFQ and provided the venous blood, 407 (50.6%) were at mid-pregnancy and 397 (49.4%) at late pregnancy. The mean gestational ages of women in mid-pregnancy and late pregnancy were 16.8 ± 1.0 weeks and 38.1 ± 1.1 weeks, respectively. The demographic characteristics of these women by stages of pregnancy are shown in Table 1. The characteristics of the women in mid- and late-term pregnancy were similar, except that women in late pregnancy were more likely to have the ethnicity, Han.

### 3.2. DHA Dietary Intake and Concentrations

Dietary DHA intake and DHA concentrations of plasma and erythrocytes are shown in Table 2. The median DHA intake, measured by the FFQ, was 18.9 mg/day for all women. The DHA intakes did not differ significantly between mid-pregnancy (18.1 mg/day) and late pregnancy (20.0 mg/day) (*p* = 0.43), while the intakes varied significantly across regions (*p* < 0.01), with the highest being in the coastland region (28.6 mg/day), followed by lakeland region (22.3 mg/day), and the lowest in the inland region (9.1 mg/day). The median DHA concentrations in plasma and erythrocytes were 2.3% and 6.3%, respectively, and differed significantly by pregnancy stage and by region (*p* < 0.01), which has been described in detail previously [28].

For agreement between DHA intake and concentration in plasma, 251 (31.2%) participants were classified into the same quartile, 334 (41.5%) into adjacent quartiles, and 54 (6.7%) were misclassified into extreme quartiles. For agreement between DHA intake and concentration in erythrocytes, 286 (35.6%) participants were classified into the same quartile, 318 (39.6%) into adjacent quartiles, and 48 (6.0%) were misclassified into extreme quartiles.

### 3.3. Correlation Analysis

The scatter plots between dietary DHA intake and its concentrations in plasma and erythrocytes are shown in Figure S2. Spearman correlation coefficients between dietary DHA intake and its concentrations are shown in Table 3. We did not observe substantial differences between crude and partial correlation coefficients, except that the coefficient in the coastland region was increased from 0.19 to 0.32. The overall partial correlation coefficients were 0.35 and 0.33 for plasma and erythrocytes. The partial correlation coefficients for women in late pregnancy (*r_s_* = 0.44 for plasma and 0.39 for erythrocytes) were slightly higher than those for women in mid-pregnancy (corresponding *r_s_* = 0.25 and 0.26) (for plasma: *z* = 3.06, *p* = 0.002; for erythrocytes: *z* = 2.06, *p* = 0.036). For plasma, the partial correlation coefficient was strongest in the coastland region (*r_s_* = 0.32), followed by the inland (*r_s_* = 0.17) and lakeland (*r_s_* = 0.16) regions. For erythrocytes, however, the correlation coefficients were stronger in the inland (*r_s_* = 0.27) and lakeland (*r_s_* = 0.23) regions than that in the coastland region, which were close to zero (*r_s_* = 0.06). As the correlation of specimen DHA and dietary intake could be affected by the use of supplements [15], we conducted the same analyses after excluding 24 women who consumed DHA supplements, and the partial correlation coefficients were not materially altered (Table S2). The demographic characteristics of these 780 women are shown in Table S3; the dietary DHA intakes and concentrations are shown in Table S4, and the corresponding scatter plots are shown in Figure S3.

**Figure 1:**
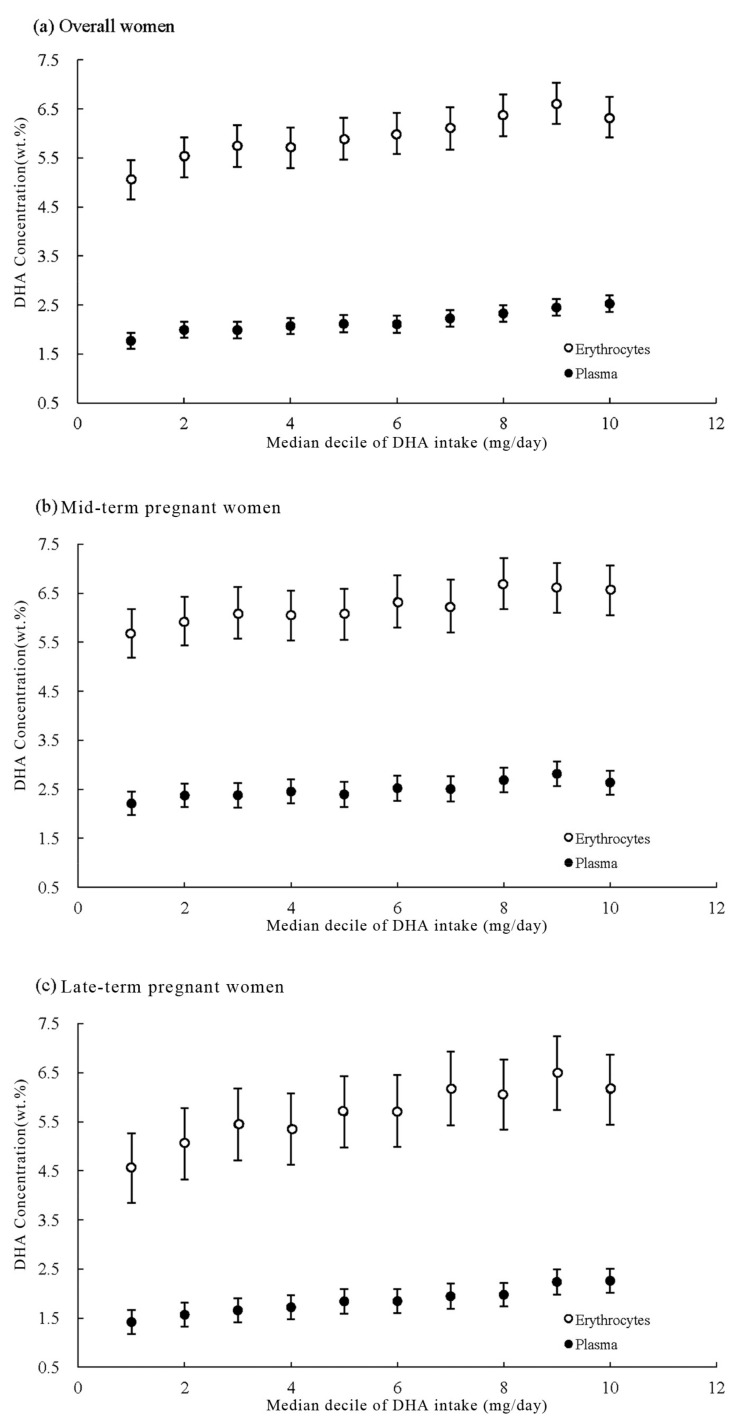
Relation between dietary DHA intake and its concentrations in plasma and erythrocytes among overall women (**a**), mid-term pregnant women (**b**) and late-term pregnant women (**c**). Deciles of DHA intake were adjusted for maternal age, pre-pregnancy body mass index, stage of pregnancy, parity, education level, ethnicity and annual family income per capita. DHA concentrations are least squares means; bars represent 95% confidence intervals. All *p* values for trends were < 0.001.

Linear trends of DHA in plasma and erythrocytes across deciles of DHA intake are presented in Figure 1. After adjustment for maternal age, pre-pregnancy BMI, stage of pregnancy, parity, education level, ethnicity and annual family income per capita, clear dose-response relations were observed between DHA intake and its concentrations in plasma as well as erythrocytes (All *p*-values for linear trends were <0.001). The least squares means of DHA concentrations increased progressively, from the first decile to the last decile of DHA intake, from 1.77% to 2.53% for plasma and from 5.06% to 6.33% for erythrocytes. The DHA concentrations of late-term pregnant women increased slightly faster than those of mid-term pregnant women, either for plasma (from 1.42% to 2.26% for late-term pregnant women, and 2.21% to 2.64% for mid-term pregnant women) or erythrocytes (from 4.45% to 6.15% and 5.68% to 6.56%, respectively).

## 4. Discussion

From this study, conducted in coastland, lakeland and inland areas of China, we observed significant positive linear correlations between dietary intake, measured with FFQ and DHA concentrations in plasma or erythrocytes, except for erythrocytes in women living in the coastland area. It is important to note that the correlations were stronger for women in late pregnancy than those in mid-pregnancy.

Several validation studies of FFQ have been conducted among pregnant women [15,16,17,18,19,20,21,31]. Although dietary DHA intake, measured by FFQ, appeared not to correlate with DHA concentrations in adipose tissue (*r_s_* = 0.01) [31], it was moderately-to-strongly correlated with the DHA content of plasma or erythrocytes, with the correlation coefficients ranging from 0.21 to 0.63 for plasma [16,17,18,21], and from 0.25 to 0.61 for erythrocytes [15,19,20]. Our overall correlation coefficients were comparable with previous studies. The Spearman partial correlation coefficients we observed between the DHA intake and its contents were 0.35 in plasma and 0.33 in erythrocytes. In addition, these correlations remained significant and positive, after excluding 24 women who consumed DHA supplements, suggesting the robustness of our results.

Notably, when we performed our analyses in subgroups stratified by stages of pregnancy, we found that the correlations were stronger among women in late pregnancy (*r_s_* = 0.44 for plasma and 0.39 for erythrocytes) than those among women in mid-pregnancy (*r_s_* = 0.25 and 0.26 for plasma and erythrocytes, respectively), which were consistent with the observations from previous studies. For plasma, the correlation coefficient was 0.21 among Brazilian women at 6–13 gestational weeks [18], whereas a relatively higher coefficient of 0.63 was observed among Canadian women at 28–35 gestational weeks [16]. For erythrocytes, the correlation coefficient was 0.25 among Norwegian women at 17–18 gestational weeks [15], whereas a relatively higher coefficient of 0.61 was observed among Australian women at 34–37 gestational weeks [19]. It is improper to make direct comparisons on the basis of these previous studies, which differed in many aspects, especially the FFQs used to measure DHA intake, the methods used to assess DHA concentration in blood specimens, population characteristics and dietary habits across countries. Yet, the differences of correlation coefficients between women in mid- and late-term pregnancy were consistently observed in our study using the same FFQ and design. 

We cannot determine whether these differences reflect a real biological phenomenon, or a combination of measurement error and small between-person variability for women in mid-pregnancy, particularly in a population whose DHA intake is low. In general, the magnitude of correlation depends on the degree of between-person variability [32]. In our study, the between-person variability in dietary DHA intake (SD = 45.9 mg/day) for women in mid-pregnancy was smaller than that for women in late pregnancy (SD = 70.1 mg/day), which might be a plausible explanation for the correlations being were stronger in late-term pregnant women than those in mid-term pregnant women. However, if the differences truly reflect a biological phenomenon, it may be partly due to the physiological and metabolic changes during pregnancy. Maternal plasma and erythrocyte DHA concentrations start to increase from very early pregnancy until 18 weeks gestation, which is unlikely of dietary origin only, but also likely reflects some maternal adaptations, including enhanced synthesis from its precursor, mobilization from maternal stores and reduced oxidation [24,25]. Thereafter, DHA status declined, with a progressively increased placental transfer of DHA to fetus [33,34], which could balance aforementioned maternal adaptations to some degree. Therefore, the correlation of maternal DHA status with dietary intake seemed stronger in late-term pregnant women.

In subgroup analyses, stratified by regions, the correlation coefficients were lower in each of three regions alone than the overall coefficients, likely due to the enlarged variation in dietary DHA intake across the regions than within the regions. Unexpectedly, the DHA concentration in erythrocytes did not reflect its intake in the coastland area, possibly due to the reduced supply of aquatic products during the closed fishing seasons when the study was conducted. DHA concentration in erythrocytes is expected to reflect long-term intake [12], while the closed fishing season led to a temporal change in dietary intake in the coastland area. Therefore, the recent one-month dietary data collected by the FFQ was likely not well reflected by the erythrocyte DHA concentration [35].

In our study, the median dietary DHA intake was highest among pregnant women residing in the coastland area (28.6 mg/day), followed by women in the lakeland area (22.3 mg/day), and lowest among those in the inland area (9.1 mg/day). Our study was conducted during the closed fishing season (from May to July), when there was a reduced supply of aquatic products, which may explain the lower DHA intake compared with the previous study of Chinese pregnant women, which reported median DHA intakes of 54.7, 51.7 and 33.3 mg/day in coastland, lakeland and inland areas, respectively [21]. The dietary DHA intakes of pregnant women in Australia [19], Norway [15], and Canada [16] were 150, 140 and 160 mg/day, respectively and were approximately three times higher than those in the coastland of China, likely due to lower consumption of fatty fish by Chinese people versus the Western population [16,36]. It is worth noting that the dietary DHA intake of pregnant women, either in China or in other countries, did not reach the consensus recommendation of 200 mg/day [8,9,10]. In our study, the average DHA intake of women in late-term pregnancy was 20.0 mg/day, which was far from enough to satisfy the fetal demand of 67 mg DHA per day for their rapid growth [37]. Our data indicated the necessity of providing pregnant women with more DHA-enriched foods, in order to cater for the extra demand of the fetus as well as the physiological needs of pregnant women [3].

Despite the inadequate DHA intake in our population, the blood DHA contents were relatively moderate. The underlying reasons are worth further investigation. One of potential reasons might be the aforementioned maternal adaptations during pregnancy [24,25]. Another reason might be our underestimation of dietary DHA intake, since other food sources of DHA such as eggs and chicken were not included in the FFQ [26]. 

The strengths of this study include the electronic FFQ with food pictures and intake reference diagrams, three study areas with contrasting dietary patterns, and the relatively large sample size. An additional strength is that we were able to assess the correlation of DHA intake with its concentration, both in mid-term pregnant women and late-term pregnant women. Besides the fact that the FFQ missed some food items containing DHA, another limitation of our study is the lack of information on cooking and food processing methods, which vary greatly in China, and may also impact the fatty acid content of food.

## 5. Conclusions

In summary, the dietary DHA intake, estimated by the semiquantitative DHA-specific FFQ was positively correlated with its concentration in plasma and erythrocytes in Chinese pregnant women, with an exception of the concentration in erythrocytes in women living in the coastland area. Our data also showed a stronger correlation in late-term pregnant women than in mid-term pregnant women. Notably, even the DHA intake among Chinese pregnant women who lived in the coastland area was not enough to satisfy the potential demands for fetal and maternal accretion [38], suggesting that Chinese pregnant women need to consume more DHA-enriched aquatic foods or take supplements, based on physician advice.

## Figures and Tables

**Table 1:** Characteristics of women in mid- and late-term pregnancy.

Characteristics	Overall (*N* = 804)		Mid-Pregnancy (*N* = 407)		Late Pregnancy (*N* = 397)		*p* Value ^1^
	*n*	%	*n*	%	*n*	%	
Age (years)							0.065
≤25	140	17.4	75	18.4	65	16.4	
>25 to 30	491	61.1	258	63.4	233	58.7	
>30	173	21.5	74	18.2	99	24.9	
Pre-pregnancy BMI (kg/m^2^)							0.235
<18.5	147	18.3	74	18.2	73	18.4	
18.5 to <25	591	73.5	293	72.0	298	75.1	
≥25	66	8.2	40	9.8	26	6.6	
Area of residence							0.904
Coastland	263	32.7	136	33.4	127	32.0	
Lakeland	267	33.2	133	32.7	134	33.8	
Inland	274	34.1	138	33.9	136	34.3	
Parity							0.317
Nulliparous	665	82.7	342	84.0	323	81.4	
Multiparous	139	17.3	65	16.0	74	18.6	
Ethnicity							0.012
Han	764	95.0	379	93.1	385	97.0	
Others	40	5.0	28	6.9	12	3.0	
Education							0.994
College or above	523	65.1	264	64.8	259	65.2	
High school	175	21.8	89	21.9	86	21.7	
Middle school or less	106	13.2	54	13.3	52	13.1	
Annual family income per capita (Yuan)							0.050
<30,000	296	36.8	151	37.1	145	36.5	
30,000 to <50,000	231	28.7	125	30.7	106	26.7	
50,000 to <100,000	191	23.8	85	20.9	106	26.7	
≥100,000	30	3.7	11	2.7	19	4.8	
Missing	56	7.0	35	8.6	21	5.3	

^1^ Chi-square test was used to compare percentages by stage of pregnancy. BMI, body mass index.

**Table 2:** Dietary docosahexanoic acid (DHA) intake and its concentrations in plasma and erythrocytes among women by pregnancy stages and regions.

	Overall	Pregnancy Stages			Regions			
		Mid-Pregnancy	Late Pregnancy	*p*-Value	Coastland	Lakeland	Inland	*p*-Value ^1^
**Dietary intake (mg/day)**								
Median	18.9	18.1	20.0	0.429	28.6	22.3	9.1	<0.001
IQR	7.8 to 45.1	7.5 to 41.8	7.9 to 47.7		15.0 to 64.4	10.4 to 44.8	2.6 to 23.0	
Adjusted median ^2^	20.2	19.4	21.7		29.1	20.3	9.3	
Adjusted IQR ^2^	8.2 to 45.1	8.3 to 42.0	9.5 to 48.4		14.6 to 57.9	10.7 to 42.6	2.7 to 21.8	
Minimum, maximum	0.0 to 850.9	0.0 to 348.8	0.0 to 850.9		0.2 to 850.9	0.0 to 348.8	0.0 to 425.6	
Mean	38.3	34.0	42.6		50.7	39.1	25.6	
SD	59.6	45.9	70.1		70.7	51.5	52.4	
**Plasma (wt. %)**								
Median	2.3	2.6	2.0	<0.001	2.8	2.2	1.9	<0.001
IQR	1.9 to 2.7	2.2 to 3.0	1.6 to 2.3		2.4 to 3.3	1.9 to 2.5	1.6 to 2.3	
Adjusted median ^2^	2.2	2.6	2.0		2.8	2.2	2.0	
Adjusted IQR ^2^	1.9 to 2.6	2.2 to 3.1	1.6 to 2.4		2.4 to 3.3	1.9 to 2.5	1.6 to 2.3	
Minimum, maximum	0.8 to 5.4	1.1 to 5.4	0.8 to 4.3		1.4 to 5.4	0.9 to 4.0	0.8 to 3.6	
Mean	2.3	2.6	2.0		2.9	2.2	2.0	
SD	0.7	0.7	0.6		0.7	0.5	0.5	
**Erythrocyte (wt. %)**								
Median	6.3	6.5	6.1	<0.001	7.5	6.3	5.5	<0.001
IQR	5.5 to 7.3	5.7 to 7.5	5.3 to 7.1		6.7 to 8.5	5.8 to 6.9	5.0 to 6.2	
Adjusted median ^2^	6.5	6.6	6.3		7.6	6.2	5.5	
Adjusted IQR ^2^	5.4 to 7.4	5.9 to 7.5	5.4 to 7.3		6.9 to 8.3	5.7 to 6.8	5.1 to 6.2	
Minimum, maximum	0.7 to 11.2	0.8 to 10.2	0.7 to 11.2		0.7 to 11.2	2.1 to 9.4	1.0 to 8.4	
Mean	6.4	6.6	6.1		7.4	6.3	5.5	
SD	1.5	1.4	1.7		1.7	1.0	1.2	

^1^ Significantly different across regions: coastland > lakeland > inland (KruskalWallis tests, followed by Bonferroni corrected Mann–Whitney tests for multiple comparisons were used). ^2^ Estimated from quartile regression models, adjusted for maternal age, pre-pregnancy body mass index, stage of pregnancy, parity, education level, ethnicity, and annual family income per capita. IQR, interquartile range; SD, standard deviation; wt. %, weight percent of total fatty acids.

**Table 3:** Spearman correlation coefficients between dietary DHA intake and its concentrations in plasma and erythrocytes.

	Plasma				Erythrocytes			
	Crude	*p*	Partial ^1^	*p*	Crude	*p*	Partial ^1^	*p*
**Overall**	0.32	<0.001	0.35	<0.001	0.34	<0.001	0.33	<0.001
**Pregnancy stages**								
Mid-pregnancy	0.27	<0.001	0.25	<0.001	0.30	<0.001	0.26	<0.001
Late pregnancy	0.45	<0.001	0.44	<0.001	0.38	<0.001	0.39	<0.001
**Regions**								
Coastland	0.19	0.007	0.32	<0.001	0.04	0.534	0.06	0.335
Lakeland	0.16	0.010	0.16	0.009	0.24	<0.001	0.23	<0.001
Inland	0.20	<0.001	0.17	0.005	0.29	<0.001	0.27	<0.001

^1^ In the overall analyses and subgroup analyses stratified by regions, we adjusted for maternal age, pre-pregnancy body mass index, stage of pregnancy, parity, education level, ethnicity, and annual family income per capita. In subgroup analyses, stratified by stages of pregnancy, we adjusted for maternal age, pre-pregnancy body mass index, parity, education level, ethnicity, and annual family income per capita.

## Supplementary Materials

The following are available online at www.mdpi.com/2072-6643/9/11/1256/s1, Figure S1: Participant flowchart. Figure S2: The scatter plots between dietary DHA intake and its concentrations in plasma and erythrocytes. Figure S3: The scatter plots between dietary DHA intake and its concentration in plasma and erythrocytes among 780 pregnant women. Table S1: Adjusted Spearman correlation coefficients between dietary DHA intake and its concentrations in plasma and erythrocytes. Table S2: Spearman partial correlation coefficients (95% confidence intervals) between dietary DHA intake and its concentrations in plasma and erythrocytes among 780 pregnant women. Table S3: Characteristics of 780 pregnant women in mid- and late pregnancy. Table S4: Dietary DHA intake and its concentrations in plasma and erythrocytes among 780 women by pregnancy stages and regions.
