# Supplementary material for: A Correlation Study of DHA Intake Estimated by a FFQ and Concentrations in Plasma and Erythrocytes in Mid- and Late Pregnancy
Source: Nutrients. 2017 Nov 16;9(11):1256. doi: 10.3390/nu9111256 (PMC5707728; doi:10.3390/nu9111256)
Supplement: Supplementary file 1 [file nutrients-09-01256-s001.zip › Supplemental files-Figure S2.pdf]

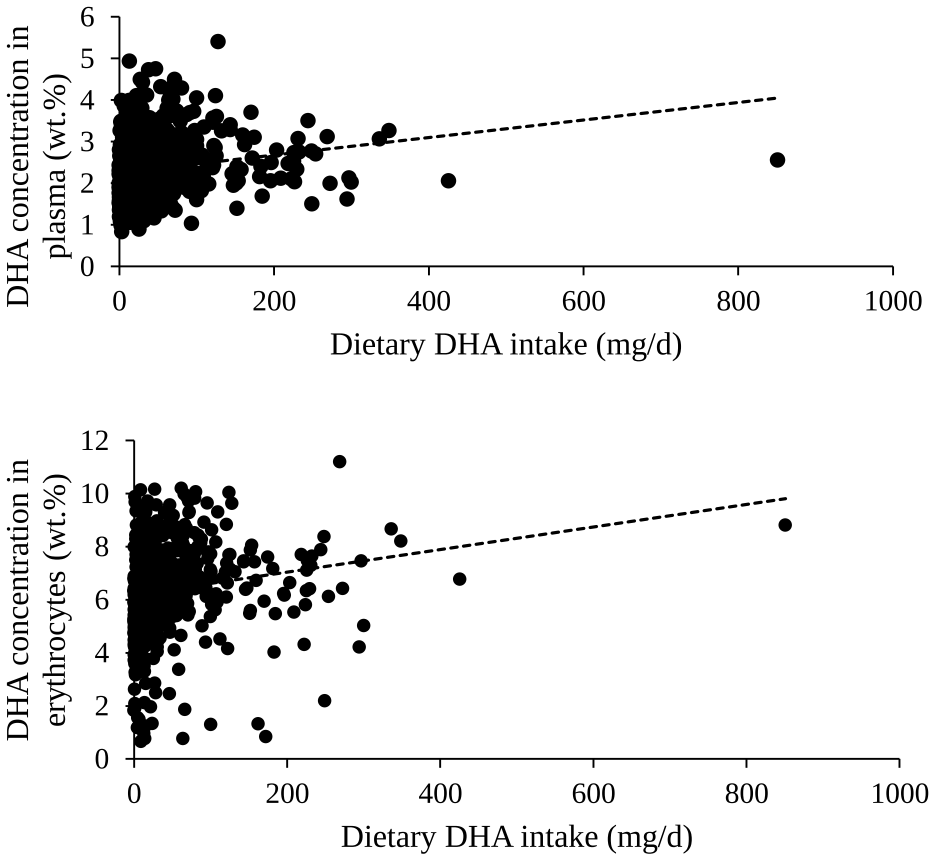

Figure S2. The scatter plots between dietary DHA intake and its concentrations in plasma and erythrocytes
