# Supplementary material for: A Correlation Study of DHA Intake Estimated by a FFQ and Concentrations in Plasma and Erythrocytes in Mid- and Late Pregnancy
Source: Nutrients. 2017 Nov 16;9(11):1256. doi: 10.3390/nu9111256 (PMC5707728; doi:10.3390/nu9111256)
Supplement: Supplementary file 1 [file nutrients-09-01256-s001.zip › Supplemental files-Tables.pdf]

Table S1. Adjusted Spearman correlation coefficients between dietary DHA intake and its concentrations in plasma and erythrocytes[1]

**Adjusted Confounders**	**Overall**	**Pregnancy stages**		**Regions**		
		Mid-pregnancy	Late pregnancy	Coastland	Lakeland	Inland
**Plasma**						
Maternal age	0.31	0.27	0.45	0.17	0.16	0.21
Pre-pregnancy body mass index	0.31	0.27	0.46	0.17	0.16	0.20
Stages of pregnancy	0.37	—	—	0.32	0.17	0.18
Parity	0.31	0.27	0.44	0.17	0.16	0.20
Education level	0.31	0.27	0.45	0.15	0.16	0.18
Ethnicity	0.31	0.26	0.46	0.17	0.15	0.19
Annual family income per capita	0.31	0.27	0.45	0.17	0.16	0.20
**Erythrocytes**						
Maternal age	0.34	0.27	0.38	0.04	0.24	0.30
Pre-pregnancy body mass index	0.34	0.28	0.39	0.04	0.24	0.29
Stages of pregnancy	0.34	—	—	0.07	0.24	0.28
Parity	0.33	0.28	0.38	0.04	0.24	0.29
Education level	0.33	0.29	0.37	0.02	0.24	0.28
Ethnicity	0.33	0.29	0.38	0.04	0.23	0.29
Annual family income per capita	0.34	0.28	0.39	0.04	0.23	0.29

[1] Individually adjusted for maternal age, pre-pregnancy body mass index, stages of pregnancy, parity, education level, ethnicity, and annual family income per capita. All P values were <0.05, except that the P values for erythrocytes in coastland were >0.05.

Table S2. Spearman partial correlation coefficients (95% confidence intervals) between dietary DHA intake and its concentrations in plasma and erythrocytes among 780 pregnant women[1]

	Plasma		Erythrocytes	
	Partial[2]	P	Partial[2]	P
**Overall**	0.35 (0.28, 0.41)	<0.001	0.33 (0.26, 0.39)	<0.001
**Pregnancy stage**				
Mid-pregnancy	0.26 (0.17, 0.35)	<0.001	0.29 (0.20, 0.38)	<0.001
Late pregnancy	0.42 (0.33, 0.50)	<0.001	0.35 (0.26, 0.44)	<0.001
**Regions**				
Coastland	0.32 (0.21, 0.43)	<0.001	0.04 (-0.08, 0.16)	0.551
Lakeland	0.12 (-0.01, 0.24)	0.063	0.21 (0.08, 0.32)	0.001
Inland	0.13 (0.01, 0.25)	0.035	0.24 (0.12, 0.35)	<0.001

[1]Among the 804 pregnant women, 24 women who consumed DHA supplements were excluded.

[2]Adjusted for maternal age, pre-pregnancy body mass index, stage of pregnancy, parity, education level, ethnicity, and annual family income per capita.

Table S3. Characteristics of the 780 pregnant women in mid- and late pregnancy.

**Characteristics**	**Overall (N=780)**		**Mid-pregnancy (N=396)**		**Late pregnancy (N=384)**		**P value[2]**
	**n**	**%**	**n**	**%**	**n**	**%**	
Age (Year)							0.034
≤25	136	17.4	74	18.7	62	16.1	
>25 to 30	477	61.2	252	63.6	225	58.6	
>30	167	21.4	70	17.7	97	25.3	
Pre-pregnancy BMI (Kg/m^2)							0.193
<18.5	140	18.0	70	17.7	70	18.2	
18.5 to <25	575	73.7	286	72.2	289	75.3	
≥25	66	8.3	40	10.1	25	6.5	

Area of residence							0.865
Coastland	257	32.9	134	33.8	123	32.0	
Lakeland	255	32.7	128	32.3	127	33.1	
Inland	268	34.4	134	33.8	134	34.9	
Parity							0.341
Nulliparous	644	82.6	332	83.8	312	81.3	
Multiparous	136	17.4	64	16.2	72	18.8	
Ethnicity							0.007
Han	741	95.0	368	92.9	373	97.1	
Others	39	5.0	28	7.1	11	2.9	
Education							0.990
College or above	505	64.7	257	64.9	248	64.6	
High school	171	21.9	86	21.7	85	22.1	
Middle school or less	104	13.3	53	13.4	51	13.3	
Annual family income per capita (Yuan)							0.024
<30 000	286	36.7	146	36.9	140	36.5	
30 000 to <50 000	227	29.1	124	31.3	103	26.8	
50 000 to <100 000	183	23.5	81	20.5	102	26.6	
≥100 000	29	3.7	10	2.5	19	5.0	
Missing	55	7.0	35	8.8	20	5.2	

[1]Among the 804 pregnant women, 24 women who consumed DHA supplements were excluded.

[2] Chi-square test was used to compare percentages by stage of pregnancy.

Table S4. Dietary DHA intake and its concentrations in plasma and erythrocytes among 780 women by pregnancy stages and regions[1]

	Overall	Pregnancy stages			Regions			
		Mid-pregnancy	Late pregnancy	P value	Coastland	Lakeland	Inland	P value[2]
**Dietary intake (mg/d)**								
Median	18.5	18.0	19.5	0.705	28.6	21.4	8.7	<0.001
IQR	7.5 to 42.2	7.5 to 40.1	7.9 to 47.7		15.1 to 63.3	10.3 to 42.4	2.6 to 20.5	
Adjusted median[3]	19.6	19.6	20.4		29.4	20.5	8.8	
Adjusted IQR[3]	7.9 to 43.8	7.9 to 43.8	8.7 to 43.9		14.4 to 57.9	10.6 to 40.5	2.7 to 19.6	
Minimum, maximum	0.0 to 348.8	0.0 to 348.8	0.0 to 296.8		0.2 to 296.8	0.0 to 348.8	0.0 to 300	
Mean	33.0	31.6	34.4		46.4	32.7	20.3	
SD	42.2	40.7	43.7		47.2	36.8	37.9	
**Plasma (wt.%)**								
Median	2.2	2.6	2.0	<0.001	2.8	2.2	1.9	<0.001
IQR	1.9 to 2.7	2.2 to 3.0	1.6 to 2.3		2.4 to 3.3	1.9 to 2.5	1.6 to 2.2	
Adjusted median[3]	2.2	2.6	2.0		2.8	2.2	2.0	
Adjusted IQR[3]	1.9 to 2.6	2.2 to 3.1	1.6 to 2.3		2.4 to 3.3	1.9 to 2.5	1.6 to 2.2	
Minimum, maximum	0.8 to 5.4	1.1 to 5.4	0.8 to 4.3		1.4 to 5.4	0.9 to 3.5	0.8 to 3.6	
Mean	2.3	2.6	2.0		2.9	2.2	1.9	
SD	0.7	0.7	0.6		0.7	0.5	0.5	
**Erythrocyte (wt.%)**								
Median	6.3	6.5	6.0	<0.001	7.5	6.3	5.5	<0.001
IQR	5.5 to 7.3	5.7 to 7.5	5.3 to 7.1		6.7 to 8.5	5.7 to 6.9	5.0 to 6.1	
Adjusted median[3]	6.4	6.5	6.2		7.5	6.3	5.5	
Adjusted IQR[3]	5.5 to 7.4	5.9 to 7.5	5.4 to 7.3		6.9 to 8.3	5.7 to 6.8	5.0 to 6.1	
Minimum, maximum	0.7 to 10.2	0.8 to 10.2	0.7 to 10.1		0.7 to 10.2	2.1 to 8.4	1.0 to 8.4	
Mean	6.3	6.6	6.1		7.3	6.2	5.4	
SD	1.5	1.4	1.7		1.7	0.9	1.2	

[1] Among the 804 pregnant women, 24 women who consumed DHA supplements were excluded.

[2] Significantly different across regions: Coastland > Lakeland > Inland (Kruskal-Wallis tests followed by Bonferroni corrected Mann-Whitney tests for multiple comparisons were used).

[3] Estimated from quantile regression models adjusted for maternal age, pre-pregnancy body mass index, stage of pregnancy, parity, education level, ethnicity, and annual family income per capita.

IQR, interquartile range; SD, standard deviation; wt.%, weight percent of total fatty acids.
